# Role of Hippocampal Lipocalin-2 in Experimental Diabetic Encephalopathy

**DOI:** 10.3389/fendo.2019.00025

**Published:** 2019-01-30

**Authors:** Anup Bhusal, Md Habibur Rahman, In-Kyu Lee, Kyoungho Suk

**Affiliations:** ^1^BK21 Plus KNU Biomedical Convergence Program, Departments of Biomedical Science and Pharmacology, School of Medicine, Kyungpook National University, Daegu, South Korea; ^2^Division of Endocrinology and Metabolism, Department of Internal Medicine, School of Medicine, Kyungpook National University, Daegu, South Korea; ^3^Brain Science and Engineering Institute, Kyungpook National University, Daegu, South Korea

**Keywords:** Lipocalin-2, diabetic encephalopathy, hippocampus, glia, neuroinflammation, cognitive dysfunction

## Abstract

Diabetic encephalopathy is a severe diabetes-related complication in the central nervous system (CNS) that is characterized by degenerative neurochemical and structural changes leading to impaired cognitive function. While the exact pathophysiology of diabetic encephalopathy is not well-understood, it is likely that neuroinflammation is one of the key pathogenic mechanisms that cause this complication. Lipocalin-2 (LCN2) is an acute phase protein known to promote neuroinflammation via the recruitment and activation of immune cells and glia, particularly microglia and astrocytes, thereby inducing proinflammatory mediators in a range of neurological disorders. In this study, we investigated the role of LCN2 in multiple aspects of diabetic encephalopathy in mouse models of diabetes. Here, we show that induction of diabetes increased the expression of both *Lcn2* mRNA and protein in the hippocampus. Genetic deficiency of *Lcn2* significantly reduced gliosis, recruitment of macrophages, and production of inflammatory cytokines in the diabetic mice. Further, diabetes-induced hippocampal toxicity and cognitive decline were both lower in *Lcn2* knockout mice than in the wild-type animals. Taken together, our findings highlight the critical role of LCN2 in the pathogenesis of diabetic encephalopathy.

## Introduction

Diabetic encephalopathy is one of the most severe microvascular complications of diabetes in the central nervous system (CNS). The measurable manifestations of diabetic encephalopathy include electrophysiological and structural changes, as well as cognitive decline (1, 2). Diabetic encephalopathy involves direct neuronal damage caused by persistent hyperglycemia, a phenomenon referred to as glucose neurotoxicity (1, 3). However, the pathogenesis of this disease is poorly understood and its diagnosis is complicated due to multiple pathogenic pathways involved (4, 5). Cognitive impairment is one of the many consequences of such toxicity that reduces the quality of life of patients (6–9). Studies on both humans and rodents have revealed a close association among hyperglycemia, oxidative stress (10–12), mitochondrial dysfunction (13, 14), and activation of inflammatory pathways (11, 15). These phenomena seem to play a crucial role in the structural and functional damage of neurons in the brain, particularly in the hippocampus, thereby leading to diabetic encephalopathy.

Increasing evidence indicates that inflammation plays an important role in the pathogenesis of diabetic encephalopathy (11, 15). Chronic diabetes initiates an inflammatory response through the activation of glial cells and the upregulation of pro-inflammatory cytokines in the hippocampus in rodent models of diabetes (16, 17). This inflammatory reaction has been suggested to lead to hippocampal neuronal loss (18, 19) and cognitive decline in diabetic mice (17). Therefore, neuroinflammation and its associated endogenous factors in the brain, particularly in the hippocampus, might be useful targets for the prevention and treatment of diabetic encephalopathy. However, molecular targets that can be used for clinical purposes have not been well-explored.

Lipocalin-2 (LCN2), also called neutrophil gelatinase-associated lipocalin (NGAL), is an acute-phase protein that has been reported to be selectively induced in a variety of tissues in diabetic mice (20, 21). LCN2 is a regulator of immune and inflammatory responses in a range of neurological diseases (22–24). Our previous studies have shown that LCN2 causes gliosis, glial activation, and increased expression of inflammatory cytokines (25–27). This inflammatory microenvironment has been correlated with neurodegenerative phenotypes in several disease models. Further, the circulating levels of LCN2 have been reported to be increased in both rodents (28) and patients with diabetes (29, 30). Based on these observations, we hypothesized that LCN2 might play an important role in the development of diabetic encephalopathy. In this study, we used mouse models of diabetes to examine the pathological role of LCN2 in the progression of diabetic encephalopathy. Our results suggest that LCN2 may contribute to the augmentation of hippocampal inflammation and subsequent pathologies associated with diabetic encephalopathy.

## Materials and Methods

### Mouse Breeding and Maintenance

Male C57BL/6 mice (Samtako, Osan, South Korea) and *Lcn2*-/- mice on pure C57BL/6 background were housed under a 12-h light/dark cycle (lights on from 07:00 to 19:00 h) at a constant ambient temperature of 23 ± 2°C with food and water provided *ad libitum*. *Lcn2*-/- mice were kindly provided by Dr. Shizuo Akira (Osaka University, Japan). *Lcn2*+/+ and *Lcn2*-/- mice were back-crossed for eight to ten generations onto the C57BL/6 background to generate homozygous animals free of background effects on the phenotypes, as previously described (31, 32). All experiments were conducted in accordance with the animal care guidelines of the National Institute of Health and efforts were made to minimize the number of animals used as well as animal suffering. Male *Lcn2* wild-type (WT, *Lcn2*+/+) and *Lcn2*-knockout (KO, *Lcn2*-/-) mice aged 8–10 weeks were used in further experiments.

### Diabetes Induction

Age-matched *Lcn2* knockout (KO, *Lcn2*-/-) and *Lcn2* wild-type (WT, *Lcn2*+/+) mice of the same background strain (C57BL/6) were used to study diabetic encephalopathy. Diabetes was induced using two experimental protocols described previously (33, 34) with slight modifications. First, multiple low-dose intraperitoneal injections of streptozotocin (STZ) dissolved in citrate buffer at pH 4.5 (MLDS; 40 mg STZ/kg body weight on 5 consecutive days) were administered to induce both pancreatic β-cytotoxic effects and STZ-specific T-cell-dependent immune reactions. Second, a single high-dose intraperitoneal injection of STZ (HDS; 150 mg/kg body weight) was administered to effect direct toxicity in pancreatic β-cells, which results in necrosis within 48–72 h and causes permanent hyperglycemia. Control mice were injected with the same volume of the citrate buffer. The day of the first STZ injection was termed day 0. Blood samples were collected from the tail vein 1 week after the injection, and glycaemia was determined using an SD CodeFreeTM glucometer (SD Biosensor Inc., Suwon, Korea). Animals with fasting blood glucose values >260 mg/dl were considered diabetic.

### High-Fat Diet Model

High-fat diet (HFD) fed mice are a robust and efficient model for type 2 diabetes and are commonly used for both mechanistic studies and as a tool for developing novel therapeutic interventions (35–37). Six-week-old male WT (C57BL/6) mice were fed a HFD in which 20% of the calories were derived from carbohydrates and 60% were derived from fat (D12492 pellets; Research Diets, Inc.). Control animals were fed an isocaloric low-fat diet/control diet (CD) in which 70% of the calories were derived from carbohydrates and 10% were derived from fat (D12450B pellets; Research Diets, Inc.). The mice were housed and maintained on a 12-hr light/dark cycle at 22 ± 2°C. Tissues were rapidly collected by sacrificing the mice according to the experimental time-points, and the samples were immediately stored at −80°C.

### Enzyme-Linked Immunosorbent Assay (ELISA)

The levels of LCN2 protein in the hippocampus, CSF, and plasma were assessed using the mouse LCN2 ELISA kit (R&D systems). The assays were performed in 96-well plates using the hippocampal tissue, CSF, or plasma (1:10 or 1:100 dilution) as per the manufacturer's protocol. Mouse recombinant LCN2 was used as a standard at concentrations ranging from 75 to 2,500 pg/ml, and the absorbance was measured at 450 and 540 nm using a microplate reader (Molecular Devices). All measurements were obtained from duplicated assays.

### Immunofluorescence Staining

Deeply anesthetized mice were sacrificed and subjected to intracardiac perfusion-fixation using a solution of 0.9% sodium chloride (VWR International, LLC) and 4% paraformaldehyde (Sigma-Aldrich) in 0.1 M phosphate-buffered saline (PBS, pH 7.4). Isolated brains were immersion-fixed in 4% paraformaldehyde for 24 h, and then incubated in 30% sucrose and embedded in optimal cutting temperature (OCT) compound for cryoprotection (Tissue-Tek; Sakura Finetek USA, Torrance, CA). Staining was carried out as described previously (27) with some modifications. Coronal brain sections (20-μm thick) were rinsed in PBS and incubated overnight with the following primary antibodies: rabbit anti-ionized calcium-binding adapter molecule 1 (Iba-1, 1:200; Wako, Osaka, Japan), rabbit anti-glial fibrillary acidic protein (GFAP, 1:1,000; Dako, Carpinteria, CA), and mouse anti-cluster of differentiation 68 (CD68, 1:200; BMA Biomedicals, Switzerland). Following incubation with primary antibodies, the sections were rinsed and incubated with fluorescein isothiocyanate (FITC)-conjugated and Cy3-conjugated secondary antibodies (1:200; Jackson ImmunoResearch, West Grove, PA). Slides were washed and then coverslipped with VECTASHIELD mounting medium (Vector Laboratories, Burlingame, CA). Images of the immunostained tissues were captured using a fluorescence microscope (Leica Microsystems, DM2500, Wetzlar, Germany).

### Cresyl Violet, 4′, 6-Diamidino-2-Phenylindole (DAPI), and Hematoxylin and Eosin (H & E) Staining

Neuronal damage was visualized using DAPI (a fluorescent chromophore that binds to double-stranded DNA in the nuclei of all cells), Cresyl violet (a Nissl stain for evaluating neuronal cell body numbers and features) (Sigma-Aldrich) (38, 39), and H & E staining. Following Cresyl violet staining, the number of pyramidal cells showing a distinct nucleus and nucleolus in each CA1 subfield of the hippocampus were counted (27, 40). For DAPI staining, sections were washed in PBS followed by mounting with VECTASHIELD mounting medium (Vector Laboratories, Burlingame, and CA) (41). Similarly, the tissues were stained with Harris' H & E solution, as described previously (42). Further, the Cresyl violet-, H & E-, and DAPI-stained tissues were visualized using bright field microscopy and fluorescent microscopy, respectively.

### Quantitative Real-Time and Traditional Reverse Transcription-Polymerase Chain Reaction (PCR)

Mice were deeply anesthetized and then perfused with normal saline through the aorta to remove the blood. Hippocampal tissues were rapidly dissected, frozen in liquid nitrogen, and homogenized in Trizol reagent (Life Technologies, Carlsbad, CA) for total RNA isolation. Total RNA (2 μg) from each sample was reverse-transcribed into cDNA using a first strand cDNA synthesis kit (MBI Fermentas, Hanover, Germany). Real-time PCR was performed using the one-step SYBR® PrimeScript TM RT-PCR kit (Perfect Real-Time; Takara Bio Inc., Tokyo) and the ABI Prism® 7000 sequence detection system (Applied Biosystems, Foster City, CA), according to the manufacturer's instructions. The 2–ΔΔCT method was used to calculate relative changes in gene expression (43), with glyceraldehyde 3-phosphate dehydrogenase (GAPDH) used as an internal control. The nucleotide sequences of the primers used in the real-time-PCR were as follows: *Lcn2*: forward, 5′-CCC CAT CTC TGC TCA CTG TC-3′; reverse, 5′-TTT TTC TGG ACC GCA TTG-3′; *Tnf-*α: forward, 5′-ATG GCC TCC TCA TCA GTT C-3′; reverse, 5′-TTG GTT TGC TAC GAC GTG-3′; *Il-6*, 5′-AGT TGC CTT CTT GGG ACT GA-3′ (forward) and 5′-TCC ACG ATT TCC CAG AGA AC-3′ (reverse) *Gapdh*: forward, 5′-TGG GCT ACA CTG AGC ACC AG-3′; reverse, 5′-GGG TGT CGC TGT TGA AGT CA-3′. Likewise, the traditional reverse transcription-PCR was performed using a DNA Engine Tetrad Peltier Thermal Cycler (MJ Research, Waltham, MA). To analyze the PCR products, 10 μl of each PCR reaction was electrophoresed on a 1% agarose gel and detected under ultraviolet (UV) light. The nucleotide sequences of the primers used in the traditional reverse transcription-PCR were as follows: *Lcn2*: forward, 5′-ATG TCA ACC TCC ACC TGG TC-3′; reverse, 5′-CAC ACT CAC CCA TTC AG-3′; *Gapdh*: forward, 5′-ACC ACA GTC CAT GCC ATC AC-3′; reverse, 5′-TCC ACC ACC CTG TTG CTG TA-3′.

### Novel Object Recognition Test

The novel object recognition (NOR) test was performed as described previously (27, 44, 45). The NOR test was performed over 3 days that included a habituation phase (5 min for 1 day), a training phase (10 min for 1 day), and a test phase (10 min for 1 day) for each mouse. The test was conducted using a metal cylinder (diameter, 7 cm; height, 10 cm) and a rectangular plastic cuboid filled with sand (5 × 5 × 10 cm). During the habituation phase, mice were allowed to acclimatize to the testing arena for 5 min in the absence of objects. During the training session, mice were exposed to two identical objects placed in opposite corners of the arena 3 cm away from the walls, and the mice were allowed to explore the objects for 10 min. During the test session, mice were placed in the arena with the familiar object in its previous location and a novel object in the place of the removed object. The total time spent exploring each object was recorded for 10 min. Exploration was defined as being within 3 cm of an object or touching it with the nose. Objects were thoroughly cleaned between trials to eliminate residual odors. The relative exploration time was measured using a discrimination index [(DI) = (time spent at the novel object—time spent at the familiar object)/(time spent at the novel object + time spent at the familiar object)]. N indicated the time spent near the novel object, while F indicated the time spent near the familiar object. Thus, a positive DI value indicates that the mice spent more time exploring the novel object than the familiar object, whereas a DI of 0 indicates that the mice spent equal amounts of time exploring the two objects.

### Y-Maze Test

The Y-maze test was performed as described previously (46) with slight modifications. Spatial cognition was examined using the spontaneous alternation task in the Y-maze apparatus. The Y-maze is a three-arm horizontal maze (length, 40 cm; width, 3 cm; wall height, 12 cm) in which the three arms are at equal angles from one another. The animals were initially placed at the center of the maze and allowed to move freely through it during a 7-min session. The series of arm entries were recorded visually; an arm entry was considered to be completed when the hind paws of the mouse were completely placed within the arm. A spontaneous alternation was defined as an entry into a different arm on each consecutive choice (i.e., ABC, CAB, or BCA, but not BAB). The arms of the maze were cleaned thoroughly with water before each trial in order to remove any residual odors. The percentage of alternations was defined as [(number of alternations)/(total arm entries)] × 100.

### Passive Avoidance Test

The step-through passive avoidance test was performed as previously described with minor modifications (47, 48). The test was performed over 3 days, including a habituation phase (day 1), a training phase (day 2), and a test phase (day 3) for each mouse. Briefly, the animals were placed in the light compartment of a two-compartment box (one light, one dark; San Diego Instruments, San Diego, CA) with the door to the dark compartment closed. Following 30 s of exploration, the door was opened. When the mouse entered the dark compartment, the door was closed and the mouse received a single electric shock (0.5 mA, 3 s) on the 2nd day. A retention test was conducted after 24 h, and the step-through latency for the animal to enter the dark compartment was recorded without the use of an electric shock. A maximum latency of 300 s was scored if the animal did not enter the dark compartment at all.

### Quantification and Statistical Analysis

For the immunohistochemical analysis, 3–4 tissue sections/animal were used to analyze microscopic images of the hippocampus. For the determination of immunofluorescence intensity, the whole image was selected, and the average intensity was measured using the ImageJ software (National Institutes of Health, Bethesda, MD). The graphs represent an average of all the images. Statistical analyses were performed with GraphPad Software (GraphPad Software, La Jolla, CA, USA). All the results have been presented as the means ± standard errors (SE). Statistical comparisons were performed using the Student's *t*-test. Differences with *p* < 0.05 (*p* < 0.05) were considered statistically significant.

## Results

### LCN2 Expression Is Increased in the Hippocampus of Diabetic Mice

**Figure 1:**
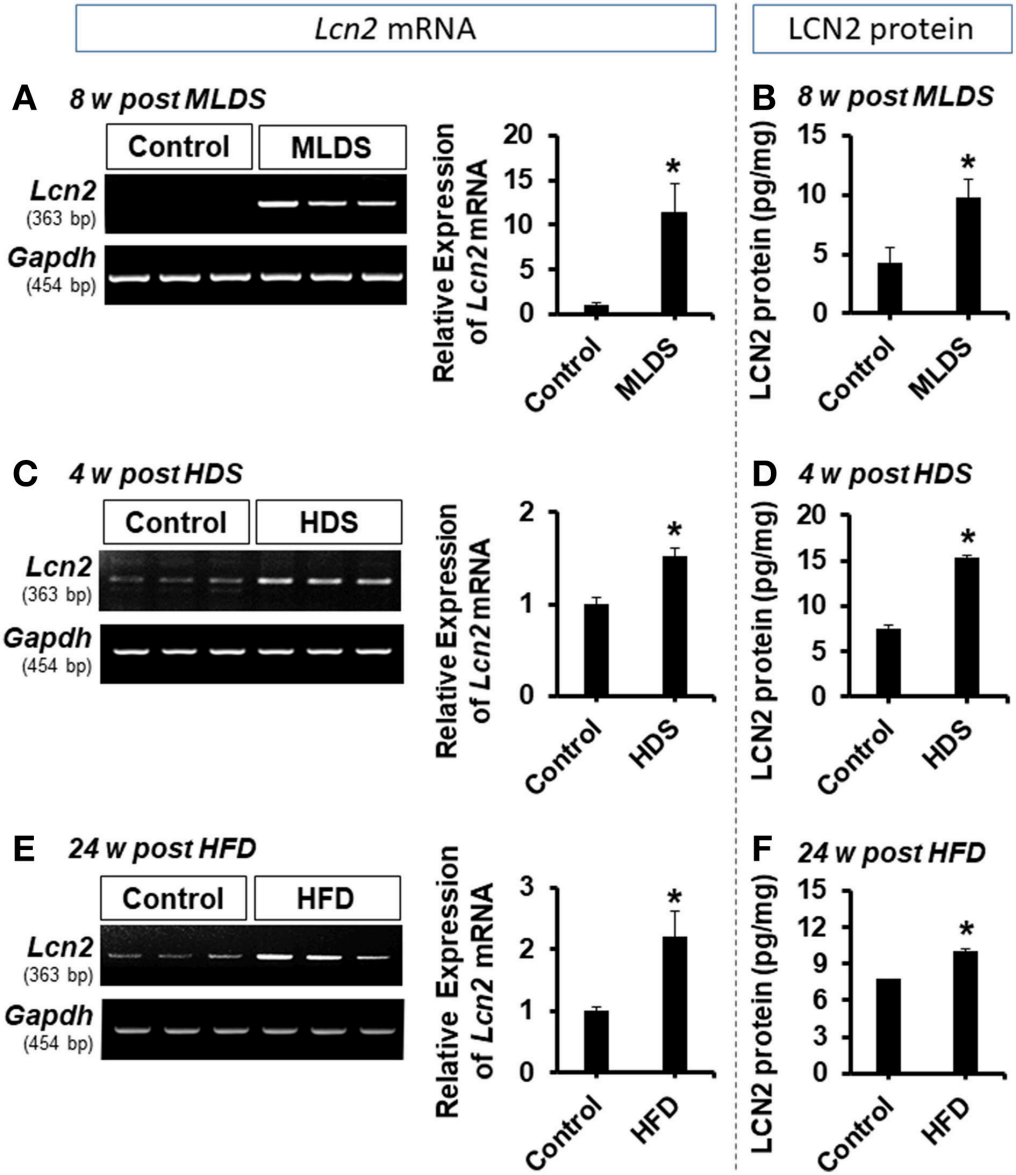
Expression of LCN2 in the hippocampus of diabetic mice. The expression of *Lcn2* mRNA in the hippocampus at 8 w post MLDS and 4 w post HDS injection was assessed by conventional PCR **(A,C)**. Further, the expression level of LCN2 protein in the hippocampus of STZ-induced diabetic mice was estimated by ELISA assay **(B,D)**. Similar upregulation of *Lcn2* mRNA and LCN2 protein was detected in the hippocampus at 24 w post HFD feeding **(E,F)**. ^*^p < 0.05 vs. the vehicle-treated control animals; Student's *t*-test; *n* = 3 for each group; data are represented as mean ± SEM. STZ, streptozotocin; MLDS, multiple low dose of STZ; HDS, high dose of STZ; HFD, high fat diet; LCN2, Lipocalin-2; w, weeks; SEM, standard error of the mean.

To study the role of LCN2 in the pathogenesis of diabetic encephalopathy, we used multiple animal models of diabetes in this study, including the multiple low dose STZ injection (MLDS), high dose STZ injection (HDS), and high fat diet (HFD) feeding models. First, intraperitoneal administration of STZ induced diabetes with elevated blood glucose levels in the first week post-injection; the increased blood glucose levels were maintained throughout the study (Supplementary Figure 1). Next, we examined the expression of *Lcn2* mRNA and protein following STZ injection using conventional RT-PCR and ELISA, respectively. We found a substantial increase in the expression of *Lcn2* mRNA in hippocampal tissues at 8 weeks post MLDS and 4 weeks post HDS administration (Figures 1A,C). Similarly, LCN2 protein level in the hippocampus was significantly increased at 8 and 4 weeks post STZ injection in both models compared to the vehicle-injected control groups (Figures 1B,D). HFD-fed mice showed a similar upregulation of *Lcn2* mRNA and increased expression of LCN2 protein in the hippocampus (Figures 1E,F). The *Lcn2* mRNA expression data was further confirmed by real time-PCR; our results were consistent with the conventional RT-PCR data (Supplementary Figures 2A–C). We predominantly used the MLDS model to investigate the role of LCN2 in the pathogenesis of diabetic encephalopathy because it represents a model of mild type 1 diabetes and closely resembles the pathophysiology observed in human patients (34, 49). The MLDS model is less toxic with a slowly progressive pancreatic β-cell death, and causes gradual elevation of blood glucose levels, minimal muscle weakness and movement abnormalities in mice, thereby providing us with an opportunity to accurately measure animal behavior. We used the HDS model to explore molecular changes and to identify the role of LCN2 in the pathogenesis of diabetic encephalopathy. Further, we also measured the LCN2 level in cerebrospinal fluid (CSF) and blood plasma of the MLDS model mice. The enzyme-linked immunosorbent assay (ELISA) revealed an increased level of LCN2 protein in the CSF and blood plasma of diabetic mice at 8 weeks post MLDS injection, when compared with the vehicle-injected control animals (Supplementary Figures 2D,E). These data suggest that induction of diabetes enhances the expression of LCN2 in the hippocampus, CSF, and blood plasma of mice.

### *Lcn2* Deficiency Reduces Diabetes-Induced Glial Activation, Proliferation, Macrophage Infiltration, and Proinflammatory Cytokine Expression in the Hippocampus

**Figure 2:**
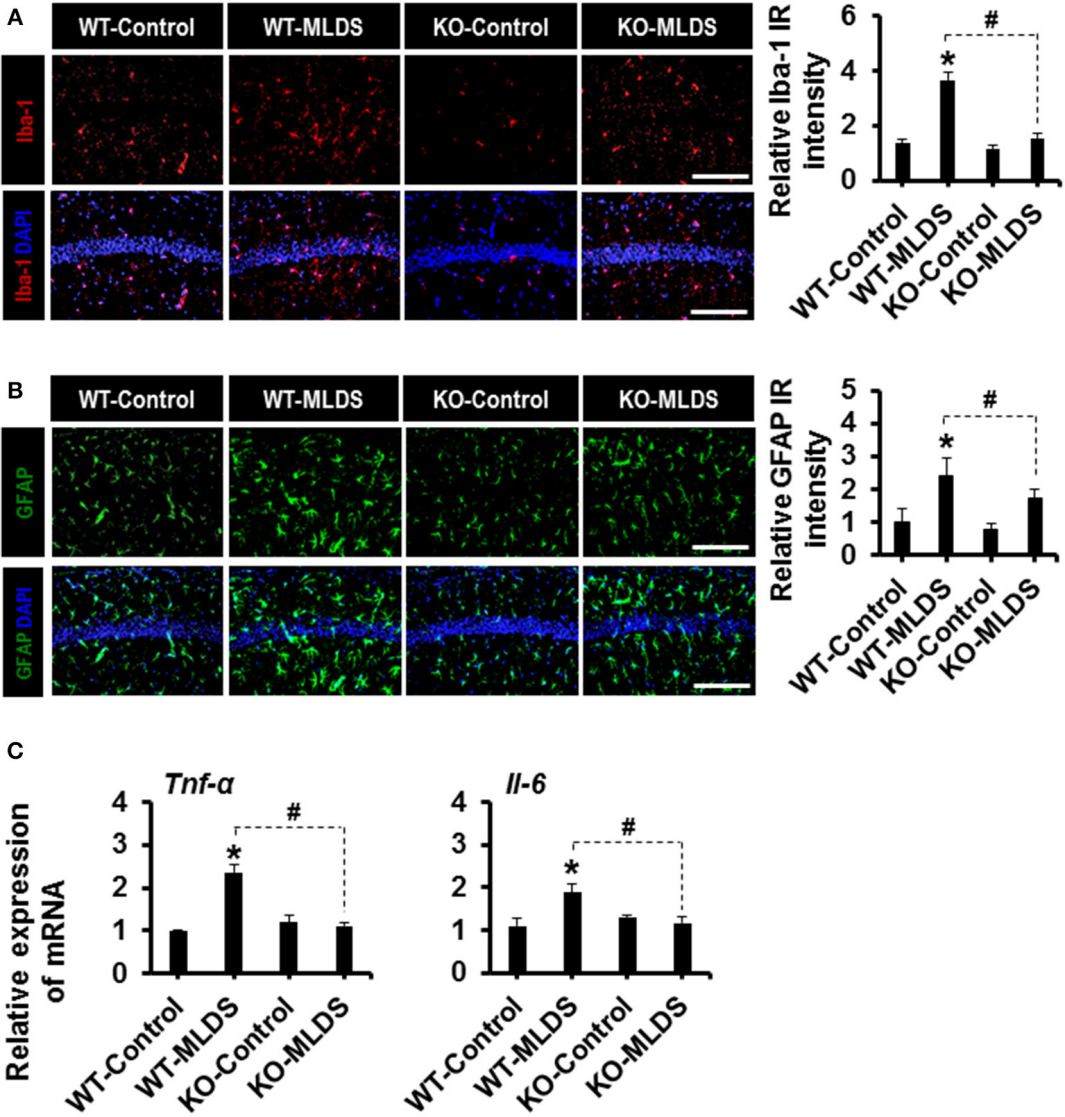
Effect of *Lcn2*-deficiency on diabetes-induced gliosis and proinflammatory cytokines in the hippocampus. Immunoreactivity (IR) of Iba-1 and GFAP was increased in the hippocampus of WT mice at 8 w post MLDS injection, whereas mice with *Lcn-2* deficiency attenuated this increase in IR **(A,B)**. The quantification of relative intensity of IR is presented adjacent to the microscopic images. The expression levels of *Tnf-*α and *Il-6* mRNAs in the hippocampus after 8 weeks of MLDS injection were evaluated by real time PCR **(C)**. ^*^p < 0.05 vs. the vehicle-treated control animals; ^#^p < 0.05 between the indicated groups; Student's *t*-test; *n* = 3 for each group; data are represented as mean ± SEM. Scale bar, 200 μm. WT, wild type; KO, knockout; MLDS, multiple low dose of STZ; w, weeks; SEM, standard error of the mean.

Glial activation and proliferation are well-known pathological features in the CNS across a range of neurological disorders (17, 50). To investigate the role of LCN2 in diabetes-induced changes in glial characteristics, brain sections were immunostained with anti-Iba-1 and anti-GFAP antibodies to label the microglia and astrocytes, respectively (51, 52). Immunofluorescence analysis showed increased immunoreactivity of Iba-1 in the hippocampus of the diabetic mice at 8 weeks (Figures 2A,B) and 4 weeks (Supplementary Figures 3A,B) post MLDS and HDS injections, respectively. We observed a significant increase in the number of Iba-1-positive microglial cells in the hippocampus of diabetic mice, where microglia exhibited enhanced Iba-1 immunoreactivity with short and thick processes when compared to control mice. These morphological features of the microglia and the increased Iba-1 immunoreactivity in the hippocampus were attenuated in the *Lcn2* KO mice. Similarly, the GFAP-positive astrocytes exhibited increased immunoreactivity and hypertrophic morphology with thick processes in the hippocampus of diabetic mice in comparison with the vehicle-injected control animals; the immunoreactivity and hypertrophic morphology were both significantly reduced in *Lcn2* KO mice. Further, the immunofluorescence-based analyses of CD68 (a macrophage marker in the brain) (53, 54) and Iba-1(a marker for both microglia and macrophages) (55, 56) in the brain tissues from diabetic mice showed a marked increase in the number of infiltrated macrophages in the hippocampus at 8 weeks post-MLDS injection compared with vehicle-injected control animals. Mice with *Lcn2*-deficiency showed a significant decrease in the number of infiltrated macrophages in the hippocampus compared with WT-diabetic animals (Supplementary Figure 4).

Furthermore, STZ-induced hyperglycemia increased the expression of *Tnf-*α and *Il-6* mRNA in the hippocampus of diabetic mice, whereas the expression levels of these cytokines were significantly attenuated in the *Lcn2*-deficient mice (Figure 2C). These findings indicate that diabetes-induced activation of glial cells and enhanced expression of proinflammatory cytokines in the hippocampus might be direct consequences of pathological inflammation, which is itself modulated by LCN2 activity (52).

### *Lcn2* Deficiency Attenuates Diabetes-Induced Loss of Hippocampal Neurons

**Figure 3:**
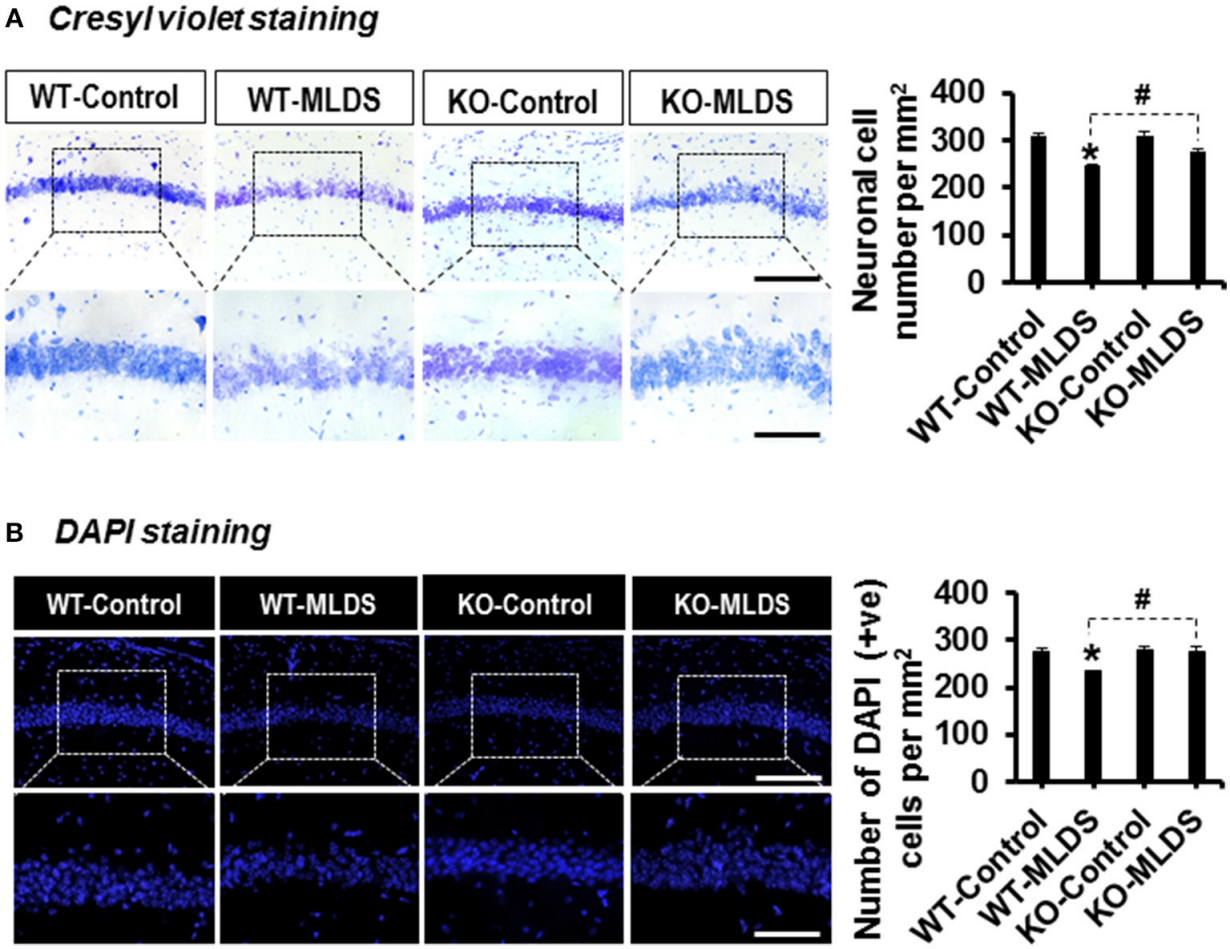
*Lcn2*-deficiency protects hippocampal neurons following diabetes induction. The number of viable neurons in the hippocampal CA1 area was determined using Cresyl violet and DAPI staining at 8 w post MLDS injection. The quantification of hippocampal neuronal number is presented adjacent to the microscopic images **(A,B)**. ^*^p < 0.05 vs. the vehicle-treated control animals; ^#^*p* < 0.05 between the indicated groups; Student's *t*-test; *n* = 3 for each group; data are represented as mean ± SEM. Scale bar, 200 and 100 μm in the original and magnified images, respectively. DAPI, 4′, 6-diamidino-2-phenylindole; WT, wild type; KO, knockout; MLDS, multiple low dose of STZ; w, weeks; +ve, positive; SEM, standard error of mean.

The increased expression of pro-inflammatory cytokines in the brain under diabetic conditions plays an important role in neuronal damage (2, 57). LCN2-mediated glial activation and subsequent inflammatory responses in the diabetic hippocampus led us to investigate the histological changes in the CA1 neurons of the hippocampus. Histological analysis using Cresyl violet and DAPI staining revealed fewer number of neurons in the CA1 of the hippocampus of diabetic mice than in that of vehicle-injected control animals. Moreover, such changes in the diabetic hippocampus were ameliorated by *Lcn2* deficiency (Figures 3A,B). Further, the H & E staining data revealed that many of the granular neurons in area CA1 of the hippocampus of WT-diabetic mice were densely stained and showed shrunken appearance with minimal or no cytoplasm compared with control animals. Fewer such degenerative features were found in the granular neurons of *Lcn2* KO mice (Supplementary Figure 5). These results indicate that the neurotoxicity in the CA1 region of the hippocampus might be related to the diabetes-induced elevation of LCN2 levels and its contribution to augmented inflammatory processes.

### Attenuation of Diabetes-Induced Cognitive Impairment in *Lcn2* KO Mice

**Figure 4:**
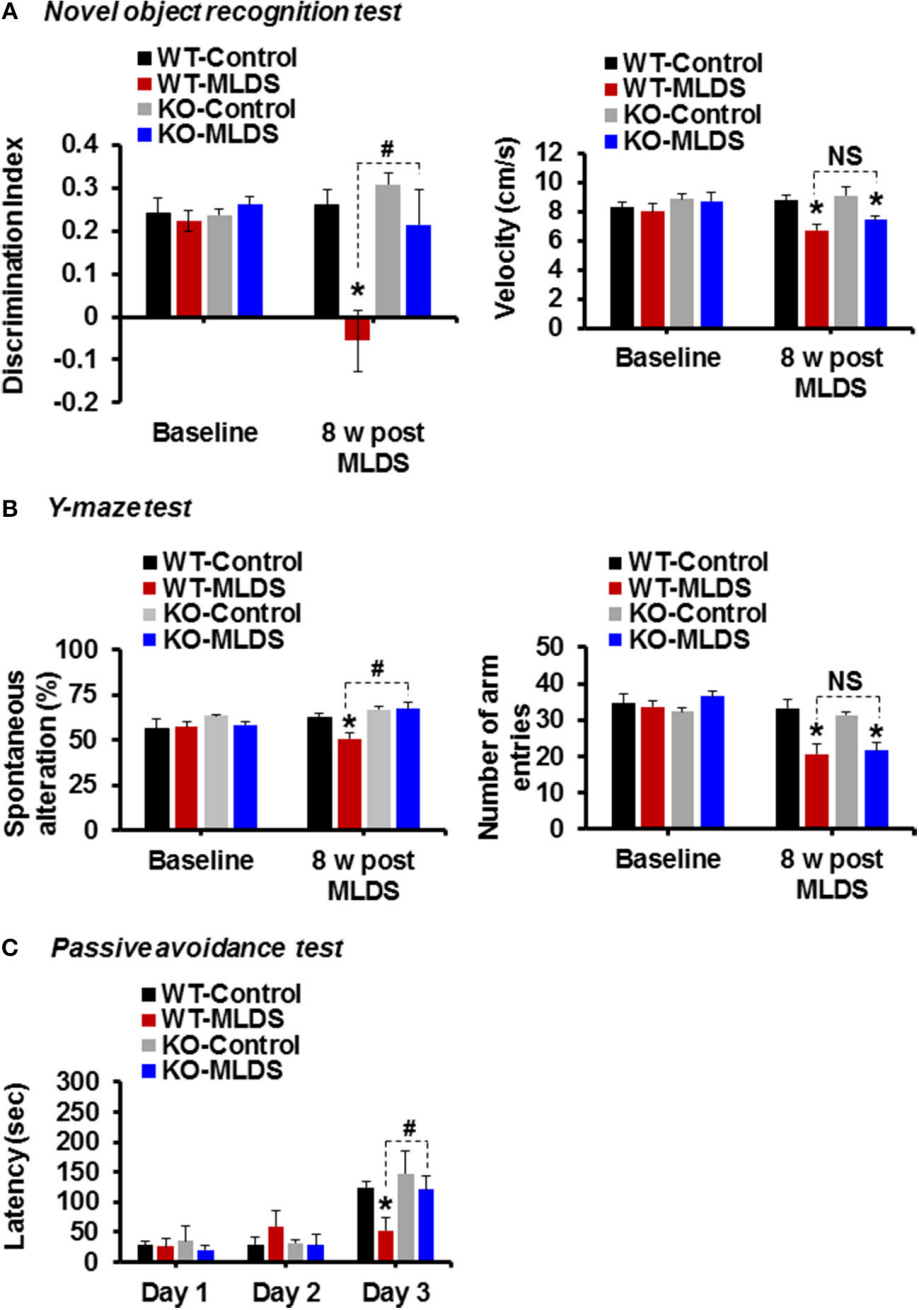
Genetic ablation of the *Lcn2* gene alleviates diabetes-induced cognitive deficits. Memory impairment in diabetic mice was measured using the novel-object recognition, Y-maze, and passive avoidance tests at 8 w post MLDS injection. The discrimination index and velocity are shown **(A)**. Spatial cognition deficit was assessed using the Y-maze test. The number of alternations and total arm entries **(B)** were compared. Cognitive impairment was further evaluated using the passive avoidance test **(C)**. ^*^p < 0.05 vs. the vehicle-treated control animals; ^#^*p* < 0.05 between the indicated groups; Student's *t*-test; *n* = 8–10 for each group; data are represented as mean ± SEM. WT, wild type; KO; knockout; MLDS, multiple low dose of STZ; w, weeks; NS, not significant; SEM, standard error of mean.

To investigate whether the cognitive function of the diabetic mice is impaired and if *Lcn2* deficiency can ameliorate this impairment, we examined the cognitive function of mice using the NOR test, which is based on differential exploration behavior with respect to familiar and new objects, and used to study short-term declarative memory and attention (58). As shown in Figure 4A, the discrimination index of the WT-diabetic mice was significantly lower than that of the vehicle-injected control animals. However, *Lcn2*-deficient diabetic mice showed an improved discrimination index compared to the WT-diabetic animals. As an alternative method to confirm whether *Lcn2* deficiency ameliorates diabetes-induced memory dysfunction, we used the Y-maze test, which measures the natural behavioral tendency of mice to explore new environments. Mice typically prefer to investigate a new arm of the maze rather than returning to one that has already been visited. Many parts of the brain, including the hippocampus, are involved in this task (46). Diabetic mice showed a decline in the percentage of spontaneous alteration compared to control animals, indicating an impairment in spatial memory function. However, *Lcn2* deficiency ameliorated this diabetes-induced memory dysfunction (Figure 4B). Further, diabetic mice of both genotypes (WT and KO) showed decrease in movement velocity and the number of arm entries at 8 weeks post-MLDS injection, which might be associated with diabetes-induced motor impairments. In line with our findings, several studies have shown a reduction in the number of total arm entries of diabetic mice compared with non-diabetic controls (59–61). Likewise, another study has found a marked reduction in speed and traveling distance of diabetic mice, indicating impairment of exploratory behavior and motor activity (62). In order to address these limitations and to confirm the changes observed in NOR and Y-maze test performances following diabetes, we performed the passive avoidance test, which is fear-aggravated, and requires lesser movement to evaluate learning and memory in rodent models of CNS disorders (48, 63). This behavior requires the association between a normally neutral environment and an aversive stimulus, which is dependent on hippocampal function. In passive avoidance test, we found that the latency during the learning trial did not differ among the experimental and control groups, indicating that all the mice had similar responses to the testing environment and the electric shock. However, as shown in Figure 4C, *Lcn2*-deficient mice showed longer latency than the WT-diabetic animals 24 h after the training process. However, we found no significant difference between the vehicle-injected control mice groups. These findings suggest that LCN2 plays a crucial role in the development of cognitive impairment associated with diabetic encephalopathy.

## Discussion

**Figure 5:**
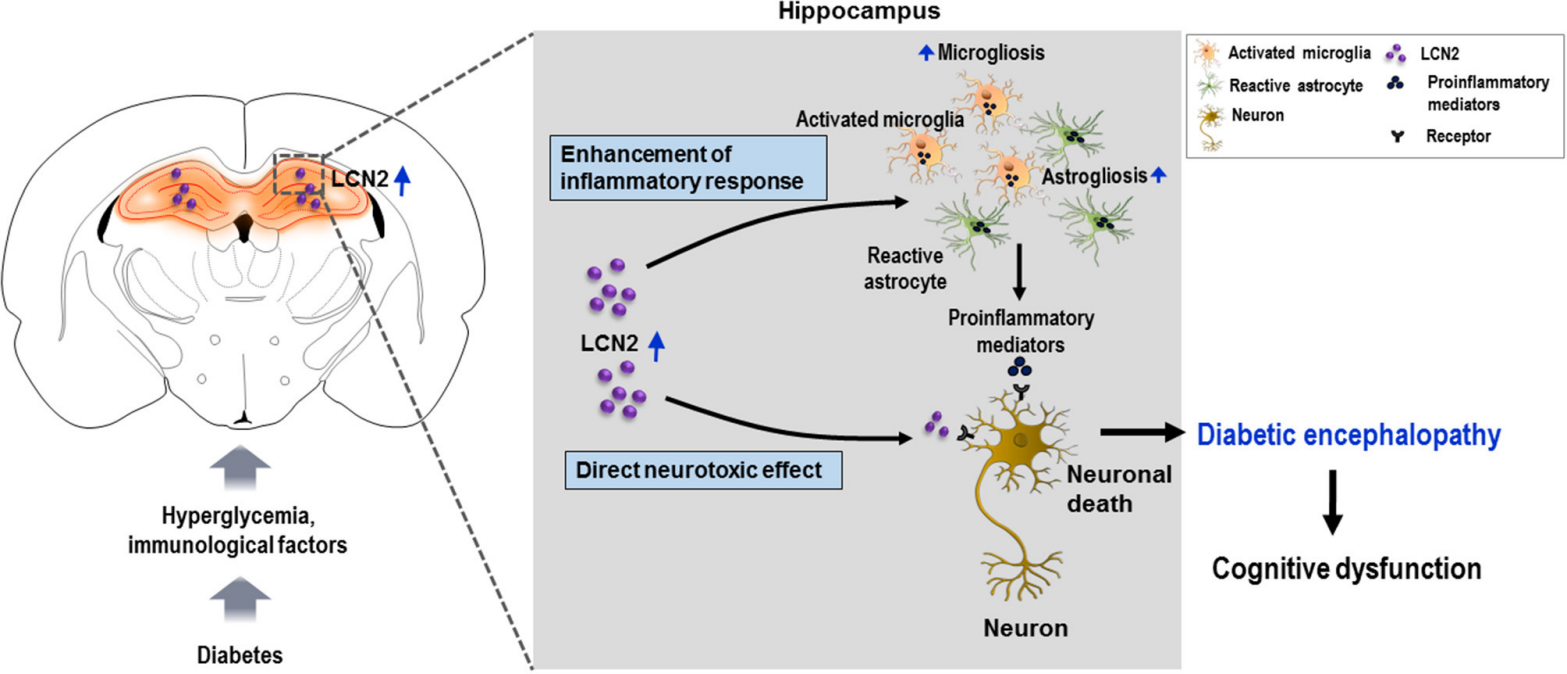
Schematic representation of the role of LCN2 in diabetic encephalopathy. Diabetes leads to the upregulation of LCN2 in the hippocampus. Induction of LCN2 enhances inflammatory activation of microglia and astrocytes, and macrophage infiltration, which causes increased expression and release of proinflammatory mediators such as *Tnf-*α and *Il-6* in the diabetic hippocampus. It is suggested that increased levels of LCN2 and proinflammatory mediators produced as a result may cause hippocampal neurotoxicity and cognitive deficits observed in diabetic mice. LCN2 may also directly induce neuronal damage by interacting with the LCN2 receptor on the surface of neurons, ultimately leading to encephalopathy in the diabetic condition. LCN2, lipocalin-2.

Diabetic encephalopathy is one of the most severe complications of diabetes mellitus that involves cognitive dysfunction and an increased incidence of dementia (64–69). Growing evidence points to the role of neuroinflammation in the progression of clinically well-recognized complications of diabetes like encephalopathy (70, 71). Several epidemiological studies have shown a strong link between poor cognitive ability and elevated inflammatory markers in patients with diabetes (72–74). Thus, targeting inflammation might be an important therapeutic strategy for the treatment of diabetic encephalopathy. Inflammation, especially in the hippocampus may lead to impairments in a variety of cognitive domains as it is the center for learning and memory processing in the brain (75, 76). The role of LCN2 in neuroinflammation and cognitive function has already been established. In the current study, we used STZ-induced diabetes and HFD mice models to evaluate the expression level of LCN2 in the hippocampus. We further employed the MLDS model to study the encephalopathy and associated cognitive impairment in diabetic mice in greater detail. We found that the induction of diabetes through STZ administration and HFD feeding significantly increased the expression of LCN2 in the hippocampus at the level of mRNA and protein. Further, deletion of the *Lcn2* gene ameliorated the diabetes-induced reactive gliosis in the hippocampus seen in the WT-diabetic mice. In addition, the hyperglycemia-induced increase in expression of pro-inflammatory cytokines was significantly attenuated in *Lcn2* KO mice. Moreover, *Lcn2*-deficient mice showed decreased neuronal loss in the CA1 region of the hippocampus following diabetes, an effect that was correlated with improved cognitive behavior in these animals. These results suggest that the diabetes-induced increase in the hippocampal LCN2 level causes neuroinflammation, which may play an important role in the development of diabetic encephalopathy and associated impairments in cognitive function (Figure 5).

Several *in vivo* studies have revealed the effect of STZ-induced diabetes on LCN2 expression in the peripheral tissues of rodents (77, 78). In these studies, STZ-induced diabetic rodents showed enhanced expression of LCN2 in the kidney and adipose tissues, respectively. However, the expression of LCN2 in the brain has not yet been studied using a mouse model of STZ-induced diabetes. To our knowledge, this is the first study to report the upregulation of LCN2 and its pathological role in the mouse hippocampus following STZ injection, which is an insulin-deficient diabetes model. As HFD-fed and STZ-injected mice are characterized by hyperinsulinemia and insulinopenic state, respectively, it is important to understand whether the presence or absence of insulin affects the expression of LCN2.

It has been reported that insulin treatment of STZ-induced diabetic mice leads to decreased LCN2 expression in skin excisional wound tissues, which has been proposed as a potential therapeutic target for improving diabetic wound healing (79). Further, HFD-fed mice show insulin resistance and hyperinsulinemia (80). In this condition, insulin cannot be used to correct the diabetic state. It has been reported that proliferator-activated receptor-γ agonist rosiglitazone, an insulin sensitizer, markedly decreases LCN2 expression in both obese mice and humans, which is correlated with a decrease in inflammation and improved insulin sensitivity (20, 28). However, upregulation of LCN2 in 3T3-L1 adipocytes by insulin treatment under hyperglycemic condition has also been reported, suggesting that glucose metabolism is required for the effect of insulin on LCN2 expression (81). In line with this, a human study revealed that circulating LCN2 level is increased after insulin intake, and also highlighted the involvement of both phosphoinositide 3-kinase and mitogen-activated protein kinase signaling pathways in insulin-induced LCN2 upregulation (82). These findings suggest a regulatory role of insulin in mediating the effects of LCN2 in metabolic diseases. Future studies may explain the mechanistic correlation between metabolic hormones and LCN2 induction with respect to neuroinflammation.

Several epidemiological studies have found increased levels of markers and mediators of inflammation in diabetic condition (83, 84). In the brain, microglia and astrocytes are the immune cells, and play a nuanced role in neuroprotection and maintenance of homeostasis (85, 86). However, under pathological conditions, such as diabetes, reactive gliosis is a well-known phenomenon (16, 87, 88). Consistently, in the present study, STZ-induced diabetes caused significant microglial and astrocytic activation 8 weeks after the injection. However, glial activation was attenuated in the *Lcn2* KO diabetic mice compared with WT mice. This result can be compared to our previous finding, in which LCN2 produced phenotypic changes in glia via Rho-associated protein kinase, and affected their migration by secreting chemokines (52). Further, LCN2 is known to act via autocrine and paracrine mechanisms to activate and recruit macrophages at the site of inflammation (89). These macrophages may further induce inflammatory mediators and exacerbate the immune response. In our study, we observed increased number of macrophages in the hippocampus of diabetic mice. A previous study using fluorescence-activated cell sorting revealed an increase in blood-brain barrier permeability, leading to macrophage infiltration in the brain of db/db mice (90). These findings suggest that the gliosis and macrophage infiltration that occur in the hippocampus of diabetic mice is likely mediated by LCN2, and that deletion of *Lcn2* prevents the inflammatory reactive gliosis and macrophage infiltration associated with encephalopathy.

Activation of immune cells in the brain of diabetic subject has been associated with several manifestations of diabetic encephalopathy (84, 91). This has been attributed to the effects of poorly controlled hyperglycemia that triggers activation of inflammatory transcription factors, such as nuclear factor kappa B, and the release of proinflammatory cytokines (91–93). LCN2 is known to stimulate various immune cells like neutrophils (94), microglia, and astrocytes (27, 95) to produce vital proinflammatory mediators, such as IL-6, IL-8, and TNF-α. In addition, the inflammatory factors released by microglia can activate astrocytes, and factors released from astrocytes may, in turn, activate microglia, further aggravating the situation (96, 97). These neurotoxic inflammatory cytokines, once released under diabetic conditions, might act directly on neurons to induce apoptosis and cell death (98, 99). In our study, WT-diabetic mice showed elevated inflammatory cytokines such as *Tnf-*α and *Il-*6 in the hippocampus, which may be due to LCN2-mediated activation of glial cells and recruitment of immune cells therein. Cognitive impairment following HFD consumption is associated with neuroinflammation impairing insulin signaling in the hippocampus of experimental animals (100). Similarly, in STZ-induced diabetic mice, hyperglycemic stress and inflammation progressively worsen the condition in the hippocampus, leading to neurodegeneration and, ultimately, diabetic encephalopathy (4). Based on these findings, it is suggested that LCN2-mediated neuroinflammation may be a potential mechanism underlying diabetic encephalopathy and cognitive deficit in both type 1 and type 2 diabetes mellitus.

Chronic neuroinflammation plays an important role in the onset and progression of various neurodegenerative diseases (101). Neurodegeneration is a condition in which neuronal structure and function are altered, leading to reduced neuronal survival and increased neuronal death in the CNS (102). Recent evidence points to an important role of LCN2 in the pathophysiology of sterile inflammatory conditions like obesity and diabetes (28, 30, 103, 104). However, LCN2 has been shown to have contradictory roles in the development of obesity or diabetes in rodents. An *in vitro* study by Zhang et al. recently demonstrated that exposure of primary adipocytes to recombinant LCN2 protein inhibits the expression of *Tnf-*α mRNA, which is suggested to be mediated by the induction of peroxisome proliferator-activated receptor gamma, a key anti-inflammatory transcription factor (105). On the contrary, Law et al. have shown that LCN2 deficiency attenuates obesity-induced expression and activity of 12-lipoxygenase and production of TNF-α in the mouse fat tissue (103). However, several studies have reported that LCN2 contributes to immune and inflammatory responses in the brain, eventually leading to the development of neurodegenerative diseases (22, 23, 106, 107). Once released, LCN2 binds to its cell-surface receptor LCN2R to regulate neuroinflammation and cell death (31, 108). In our study, Cresyl violet and DAPI staining revealed significant neuronal loss in the CA1 region of the hippocampus of diabetic WT mice compared to *Lcn2* KO mice, which is consistent with other studies (27, 41). Further, this result was supported by H & E staining. These results suggest that elevated levels of LCN2 in the hippocampus may potentiate neuroinflammation and cause neuronal loss following diabetes.

Various studies indicate that neuroinflammation and neuronal cell death are implicated in diabetes-associated learning and memory deficits (57, 62). Previously, our research group has reported increased LCN2 levels in patients with cognitive impairment (109); these findings are consistent with the study by Dekens et al. (110). In this study, WT-diabetic mice showed poor cognitive function when compared with *Lcn2* KO mice as measured by the NOR, Y-maze, and passive avoidance tests. In chronic conditions such as diabetes, over-secretion of LCN2 in the hippocampus may subsequently aggravate the neural imbalance in the hippocampus, and lead to impaired cognitive function. Our previous findings of LCN2 treatment inducing inflammatory activation of glial cells and having a toxic effect on co-cultured hippocampal neurons supports this notion (27). Other groups have reported a specific role of LCN2 in the decline of cognitive function (111) and suggested that its upregulation following stress reduces dendritic spine density in hippocampal neurons (112). Taken together, these findings suggest that LCN2 can mediate neuroinflammation via activation of glial cells and increased expression of inflammatory cytokines which leads to hippocampal neuronal death and impairs cognitive function across domains in patients with diabetes (Figure 5). Thus, LCN2 upregulation in the hippocampus might be a potential pathogenic mechanism leading to further disruption of the hippocampus through neuroinflammation in the diabetic state. Increased level of circulating LCN2 has been considered an inflammatory marker closely associated with insulin resistance and hyperglycemia in patients with diabetes (20). Therefore, the control of hyperglycemia-induced expression of LCN2 or its activity in the hippocampus may be important for neuroprotection in these patients.

In summary, our findings suggest that increased LCN2 expression due to diabetes is critical for the development of several manifestations of diabetic encephalopathy, in which LCN2-mediated inflammatory reaction as well as direct toxicity by interacting with its receptors in the hippocampal neurons of diabetic animals has been proposed as a potential mechanism. The extent to which it can worsen cognitive ability in diabetic mice makes LCN2 a promising target for therapeutic interventions against diabetic encephalopathy.

## Ethics Statement

This study was carried out in accordance with the recommendations of Kyungpook National University Animal Care Committee. The protocol was approved by Kyungpook National University Animal Care Committee.

## Author Contributions

All authors have made a substantial intellectual contribution to this work, and approved submission of the manuscript. AB and MR designed and performed the research, analyzed the data, and prepared the manuscript. I-KL provided essential reagents. KS directed the study and was involved in all aspects of the experimental design, data analysis, and manuscript preparation.

### Conflict of Interest Statement

The authors declare that the research was conducted in the absence of any commercial or financial relationships that could be construed as a potential conflict of interest.

## Supplementary Material

Supplementary Figure 1 Blood glucose levels in STZ-induced diabetic mice. After intraperitoneal injection of low dose of STZ (MLDS), blood glucose levels significantly increased within the first week and remained significantly high throughout the course of the study as compared to control non-diabetic animals. ^*^*p* < 0.05 vs. the vehicle-treated control animals; Student's *t*-test; *n* = 4–6 for each group; data are represented as mean ± SEM. WT, wild type; KO; knockout; MLDS, multiple low dose of STZ; w, weeks; SEM, standard error of mean.

Supplementary Figure 2 LCN2 expression following diabetes induction. The expression of *Lcn2* mRNA in the hippocampus at 8 w of MLDS **(A)** and 4 w of HDS **(B)** injection, and 24 w post HFD feeding **(C)** was assessed using real-time PCR. Further, the expression levels of LCN2 protein in the CSF **(D)** and blood plasma **(E)** from diabetic mice were estimated using ELISA. ^*^*p* < 0.05 vs. the vehicle-treated control animals; Student's *t*-test; *n* = 3 for each group; data are represented as mean ± SEM. MLDS, multiple low dose of STZ; HDS, high dose of STZ, HFD, high fat diet; LCN2, Lipocalin-2; PCR, polymerase chain reaction; CSF, cerebrospinal fluid; ELISA, enzyme-linked immunosorbent assay; w, weeks; SEM, standard error of mean.

Supplementary Figure 3 Effect of *Lcn2*-deficiency on diabetes (HDS)-induced gliosis in the hippocampus. Immunoreactivity (IR) of Iba-1 and GFAP was increased in the hippocampus of WT mice after 4 w of HDS injection, whereas *Lcn-2* deficiency attenuated such an increase in IR **(A,B)**. The quantification of relative intensity of IR is presented adjacent to the microscopic images. ^*^*p* < 0.05 vs. the vehicle-treated control animals; #*p* < 0.05 between the indicated groups; Student's *t*-test; *n* = 3 for each group; data are represented as mean ± SEM. Scale bar, 200 μm (Iba-1 stained images) and 100 μm (GFAP-stained images). WT, wild-type; KO, knockout; NS, not significant; HDS, high dose of STZ; w, weeks; SEM, standard error of mean; Iba-1, ionized calcium-binding adapter molecule 1; GFAP, glial fibrillary acidic protein.

Supplementary Figure 4 Macrophage infiltration in the hippocampus of diabetic mice. To determine the role of *Lcn2*-deficiency in diabetes-induced infiltration of macrophages in the hippocampus, co-immunostaining of CD68 (red) and Iba-1 (green) was performed using brain tissue sections collected from WT and *Lcn2* KO mice at 8 w post-MLDS/vehicle injection. Quantification of CD68^+^/Iba-1^−^ cells (macrophages) is presented adjacent to the representative images. Arrows indicate the CD68^+^ macrophages. ^*^*p* < 0.05 vs. vehicle-treated control animals, #*p* < 0.05 between the indicated groups, Student's *t*-test; *n* = 3 for each group; data are presented as mean ± SEM. Scale bar, 200 μm. CD68, cluster of differentiation 68; Iba-1, ionized calcium-binding adapter molecule 1; WT, wild type; KO, knockout; MLDS, multiple low doses of streptozotocin; w, weeks; SEM, standard error of the mean.

Supplementary Figure 5 *Lcn2*-deficiency confers protection against diabetes-induced degeneration of hippocampal granular neurons. To determine the role of LCN2 in diabetes-induced loss of hippocampal tissue integrity and neuronal degeneration, H and E staining was performed using brain tissue sections collected from WT and *Lcn2* KO mice at 8 w post-MLDS/vehicle injection. Quantification of percentage of degenerated neurons in the hippocampus is presented adjacent to the representative images. ^*^*p* < 0.05 vs. vehicle-treated control animals, #*p* < 0.05 between the indicated groups, Student's *t*-test; *n* = 3 for each group; data are presented as mean ± SEM. Scale bar, 200 and 100 μm in the original and magnified images, respectively. LCN2, lipocalin-2; H and E, hematoxylin and eosin; WT, wild type; KO, knockout; MLDS, multiple low doses of streptozotocin; w, weeks; SEM, standard error of mean.
