# Corrigendum: Role of Hippocampal Lipocalin-2 in Experimental Diabetic Encephalopathy

**DOI:** 10.3389/fendo.2019.00239

**Published:** 2019-04-09

**Authors:** Anup Bhusal, Md Habibur Rahman, In-Kyu Lee, Kyoungho Suk

**Affiliations:** ^1^BK21 Plus KNU Biomedical Convergence Program, Departments of Biomedical Science and Pharmacology, School of Medicine, Kyungpook National University, Daegu, South Korea; ^2^Division of Endocrinology and Metabolism, Department of Internal Medicine, School of Medicine, Kyungpook National University, Daegu, South Korea; ^3^Brain Science and Engineering Institute, Kyungpook National University, Daegu, South Korea

**Keywords:** Lipocalin-2, diabetic encephalopathy, hippocampus, glia, neuroinflammation, cognitive dysfunction

In the original article, there was a mistake in Figure 1 as published. The *Gapdh* band image used in Figure 1C was from the pilot experiment. The corrected Figure 1 appears below.

**Figure 1:**
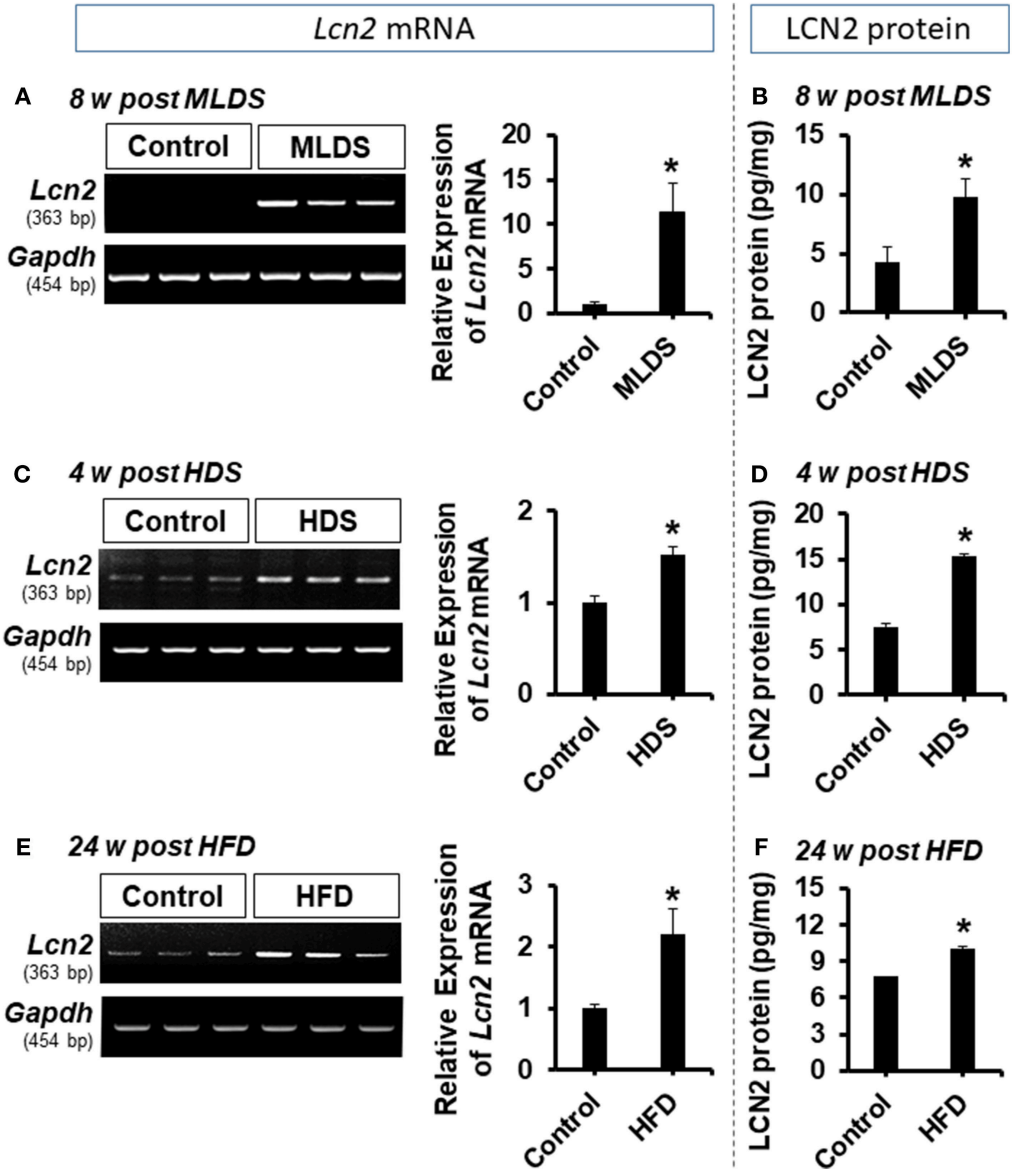
Expression of LCN2 in the hippocampus of diabetic mice. The expression of *Lcn2* mRNA in the hippocampus at 8 w post MLDS and 4 w post HDS injection was assessed by conventional PCR **(A,C)**. Further, the expression level of LCN2 protein in the hippocampus of STZ-induced diabetic mice was estimated by ELISA assay **(B,D)**. Similar upregulation of *Lcn2* mRNA and LCN2 protein was detected in the hippocampus at 24 w post HFD feeding **(E,F)**. ^*^p < 0.05 vs. the vehicle-treated control animals; Student's *t*-test; *n* = 3 for each group; data are represented as mean ± SEM. STZ, streptozotocin; MLDS, multiple low dose of STZ; HDS, high dose of STZ; HFD, high fat diet; LCN2, Lipocalin-2; w, weeks; SEM, standard error of the mean.

The authors apologize for this error and state that this does not change the scientific conclusions of the article in any way. The original article has been updated.

