# Supplementary figures and images for: Role of Hippocampal Lipocalin-2 in Experimental Diabetic Encephalopathy
Source: Front Endocrinol (Lausanne). 2019 Jan 30;10:25. doi: 10.3389/fendo.2019.00025 (PMC6363678; doi:10.3389/fendo.2019.00025)

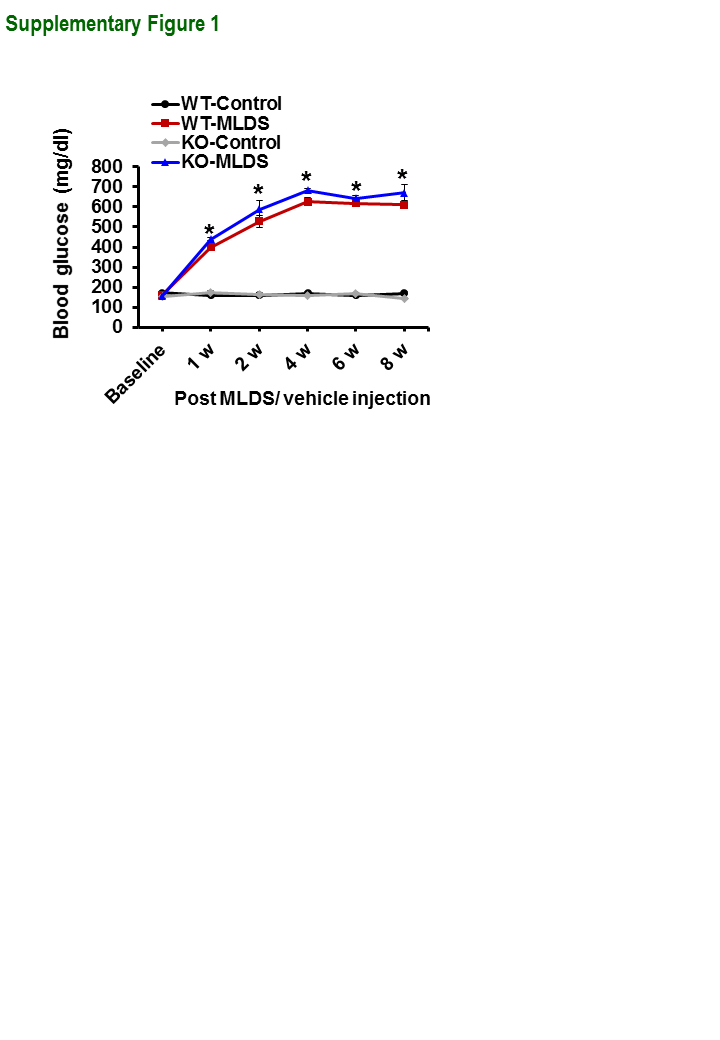

Supplement: Supplementary Figure 1 — Blood glucose levels in STZ-induced diabetic mice. After intraperitoneal injection of low dose of STZ (MLDS), blood glucose levels significantly increased within the first week and remained significantly high throughout the course of the study as compared to control non-diabetic animals. *p < 0.05 vs. the vehicle-treated control animals; Student's t-test; n = 4–6 for each group; data are represented as mean ± SEM. WT, wild type; KO; knockout; MLDS, multiple low dose of STZ; w, weeks; SEM, standard error of mean. [file Image_1.TIF]

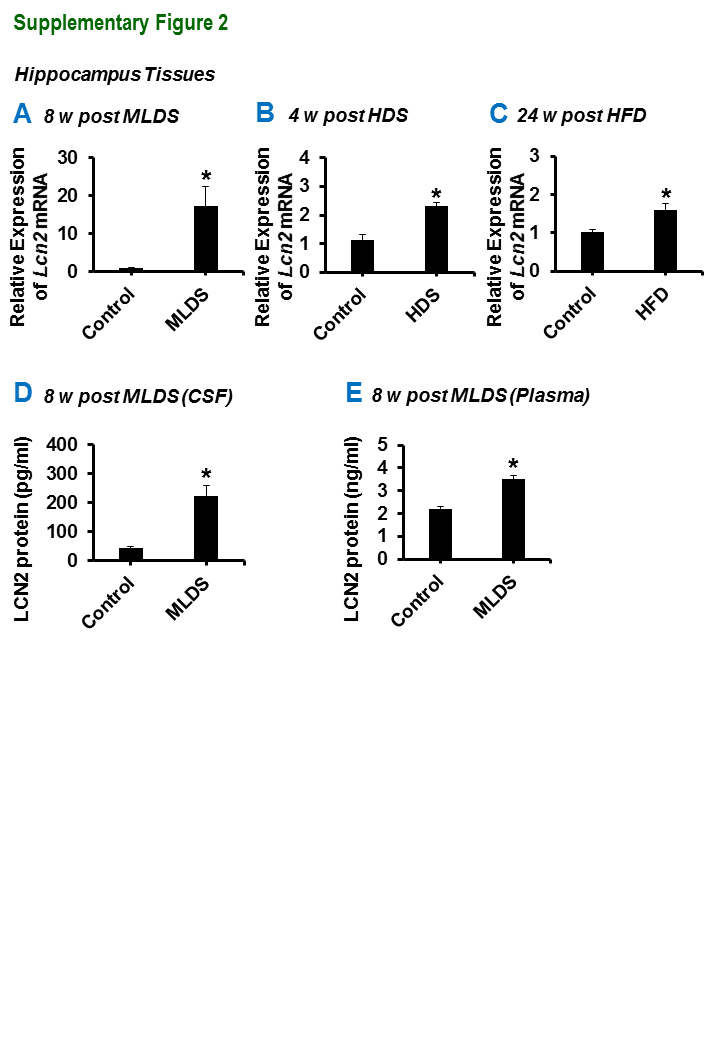

Supplement: Supplementary Figure 2 — LCN2 expression following diabetes induction. The expression of Lcn2 mRNA in the hippocampus at 8 w of MLDS (A) and 4 w of HDS (B) injection, and 24 w post HFD feeding (C) was assessed using real-time PCR. Further, the expression levels of LCN2 protein in the CSF (D) and blood plasma (E) from diabetic mice were estimated using ELISA. *p < 0.05 vs. the vehicle-treated control animals; Student's t-test; n = 3 for each group; data are represented as mean ± SEM. MLDS, multiple low dose of STZ; HDS, high dose of STZ, HFD, high fat diet; LCN2, Lipocalin-2; PCR, polymerase chain reaction; CSF, cerebrospinal fluid; ELISA, enzyme-linked immunosorbent assay; w, weeks; SEM, standard error of mean. [file Image_2.TIF]

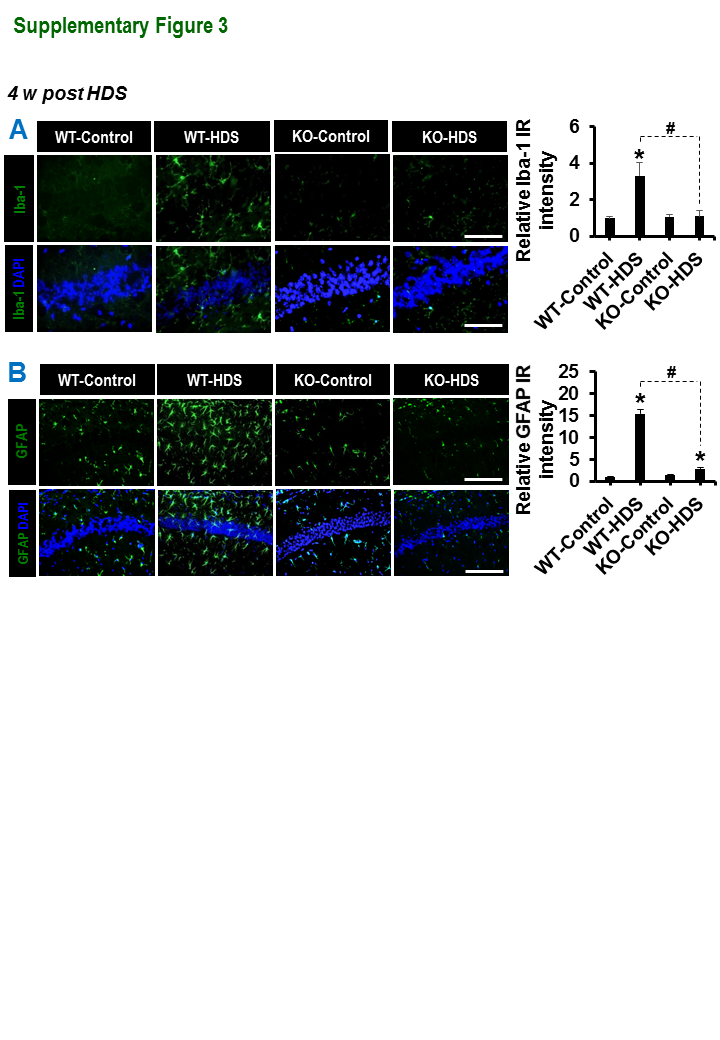

Supplement: Supplementary Figure 3 — Effect of Lcn2-deficiency on diabetes (HDS)-induced gliosis in the hippocampus. Immunoreactivity (IR) of Iba-1 and GFAP was increased in the hippocampus of WT mice after 4 w of HDS injection, whereas Lcn-2 deficiency attenuated such an increase in IR (A,B). The quantification of relative intensity of IR is presented adjacent to the microscopic images. *p < 0.05 vs. the vehicle-treated control animals; #p < 0.05 between the indicated groups; Student's t-test; n = 3 for each group; data are represented as mean ± SEM. Scale bar, 200 μm (Iba-1 stained images) and 100 μm (GFAP-stained images). WT, wild-type; KO, knockout; NS, not significant; HDS, high dose of STZ; w, weeks; SEM, standard error of mean; Iba-1, ionized calcium-binding adapter molecule 1; GFAP, glial fibrillary acidic protein. [file Image_3.TIF]

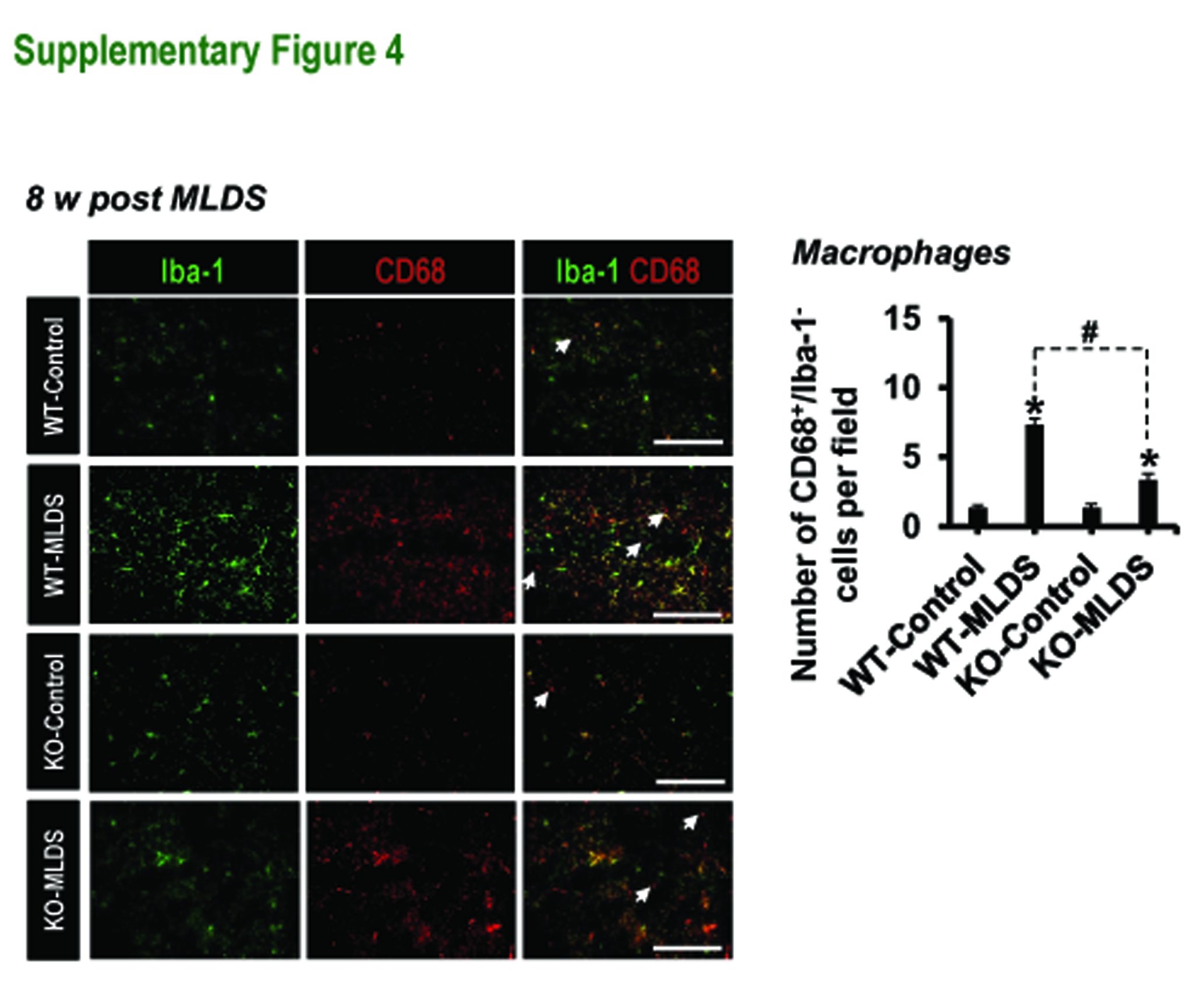

Supplement: Supplementary Figure 4 — Macrophage infiltration in the hippocampus of diabetic mice. To determine the role of Lcn2-deficiency in diabetes-induced infiltration of macrophages in the hippocampus, co-immunostaining of CD68 (red) and Iba-1 (green) was performed using brain tissue sections collected from WT and Lcn2 KO mice at 8 w post-MLDS/vehicle injection. Quantification of CD68+/Iba-1− cells (macrophages) is presented adjacent to the representative images. Arrows indicate the CD68+ macrophages. *p < 0.05 vs. vehicle-treated control animals, #p < 0.05 between the indicated groups, Student's t-test; n = 3 for each group; data are presented as mean ± SEM. Scale bar, 200 μm. CD68, cluster of differentiation 68; Iba-1, ionized calcium-binding adapter molecule 1; WT, wild type; KO, knockout; MLDS, multiple low doses of streptozotocin; w, weeks; SEM, standard error of the mean. [file Image_4.TIF]

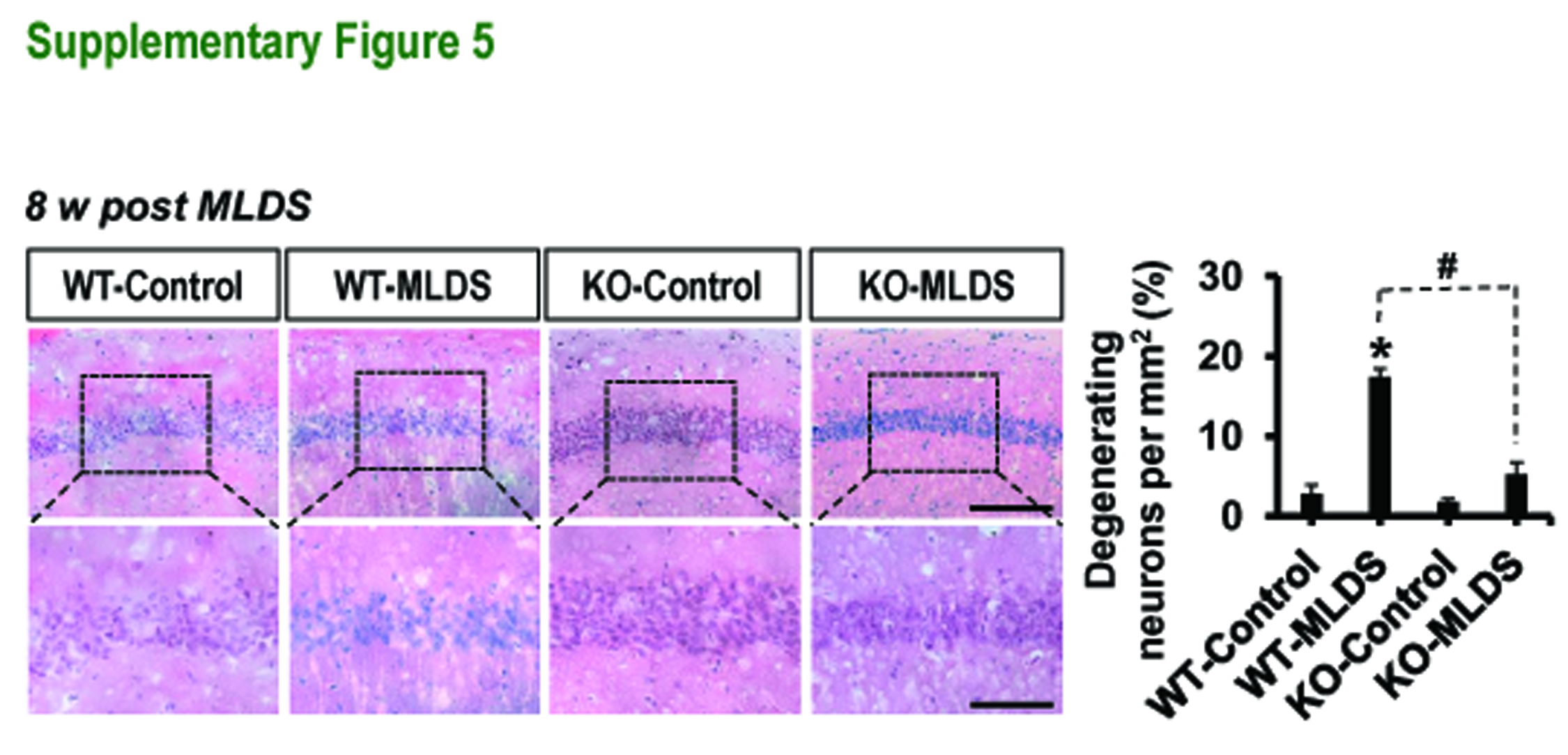

Supplement: Supplementary Figure 5 — Lcn2-deficiency confers protection against diabetes-induced degeneration of hippocampal granular neurons. To determine the role of LCN2 in diabetes-induced loss of hippocampal tissue integrity and neuronal degeneration, H and E staining was performed using brain tissue sections collected from WT and Lcn2 KO mice at 8 w post-MLDS/vehicle injection. Quantification of percentage of degenerated neurons in the hippocampus is presented adjacent to the representative images. *p < 0.05 vs. vehicle-treated control animals, #p < 0.05 between the indicated groups, Student's t-test; n = 3 for each group; data are presented as mean ± SEM. Scale bar, 200 and 100 μm in the original and magnified images, respectively. LCN2, lipocalin-2; H and E, hematoxylin and eosin; WT, wild type; KO, knockout; MLDS, multiple low doses of streptozotocin; w, weeks; SEM, standard error of mean. [file Image_5.TIF]
